# The implementation of computer-aided detection in an initial endoscopy training improves the quality measures of trainees’ future colonoscopies: a retrospective cohort study

**DOI:** 10.1007/s00464-025-11890-3

**Published:** 2025-06-30

**Authors:** Zofia Orzeszko, Tomasz Gach, Sandra Necka, Kajetan Ochwat, Piotr Major, Mirosław Szura

**Affiliations:** 1 Department of Surgery, Faculty of Health Sciences, Jagiellonian University, Trynitarska 11 Street, 31-061 Krakow, Poland; 2 School of Medicine, Jagiellonian University Medical College, Krakow, Poland; 3 2nd Department of General Surgery, Jagiellonian University Medical College, Krakow, Poland

**Keywords:** Artificial intelligence (AI), Computer-aided detection (CADe), Endoscopy training, Adenoma detection rate (ADR)

## Abstract

**Introduction:**

The implementation of computer-aided detection (CADe) systems has resulted in a growing number of young endoscopists being trained using AI-enhanced devices. The potential impact of AI-enhanced training on the trainees’ future performance is undefined. This study aimed to evaluate the quality indicators of endoscopists trained in an AI environment compared to those trained conventionally.

**Methods and procedure:**

In this retrospectively study, the independent performance of six endoscopists was evaluated after they had undergone initial training using either CADe (group A) or conventional endoscopy (group B: without CADe). Quality indicators and detection rates of laterally spreading tumors (LSTs) were compared between the two groups.

**Results:**

A total of 6000 patients were included in the analysis. Groups were equal demographically and had similar cecal intubation rate. Withdrawal time (WT) was longer in the AI-trained group (mean difference 0.8 min; 95% confidence interval [CI]: 0.6–1.0). AI-trained group had also a significantly improved adenoma detection rate (ADR) by 5.3% (95% CI: 2.9–7.6%) and sessile lesion detection rate (SDR) by 5.4% (95% CI: 3.8–7.0%). AI-assisted training enhanced the detection of non-granular LSTs smaller than 20 mm by 0.2% (95% CI 0.1% to 0.4%) and was identified as a factor of high-quality performance in terms of ADR and SDR (OR 1.27, 95% CI: 1.14–1.42; OR 1.93, 95% CI: 1.10 to 3.37, respectively).

**Conclusions:**

Endoscopists trained in colonoscopy using AI exceeded the aspirational targets of the quality guidelines when those trained conventionally achieved minimum quality measures.

**Graphical abstract:**

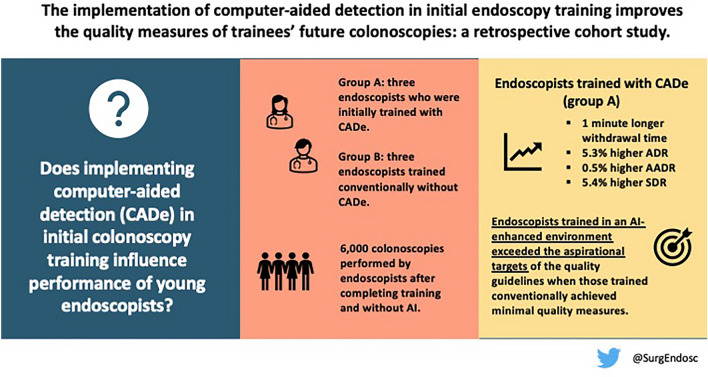

Artificial intelligence (AI) is consistently revolutionizing the medical field. The potential applications of AI in endoscopy have not yet been fully recognized and represent a dynamic area of research and development. Colorectal cancer (CRC) remains a significant global health burden and the second-leading cause of cancer-related deaths in both the United States and worldwide [1]. Colonoscopy is the gold standard for CRC screening, which is essential in detecting and removing preneoplastic and neoplastic lesions [2]. Specific performance measures were developed which are addressed in quality improvement initiatives and remain the main target of technological improvement [3, 4]. The variability in detecting preneoplastic lesions remains a significant limitation of cancer prevention [5].

Adenoma detection rate (ADR) is an independent predictor of the risk of interval or post-colonoscopy colorectal cancer (PC-CRC) [6–9]. In the control groups of randomized controlled trials, the heterogeneity of ADR ranged from 8.2% to 68.1% [10]. Approximately 26% of colorectal adenomas and 9% of advanced adenomas are missed on a single colonoscopy [11]. Missed polyps might be in areas not visible on-screen due to bowel anatomy or appear on-screen but remain unrecognized by the endoscopist. Both reasons are addressed by novel technologies [12–14].

Recent advances in AI technology have highlighted its valuable role in improving colonoscopy procedures, leading to its growing use in clinical practice. Computer-aided detection (CADe) based on AI may improve the colonoscopy quality. Several studies have shown that CADe significantly lowered miss rates and increased ADR [15–19]. The improvement in ADR was noted for both expert and non-expert attending endoscopists [20, 21]. Although the improved detection of benign adenomas does not extend to advanced adenomas, the implementation of CADe in screening colonoscopy was proven to be a cost-saving strategy to prevent colorectal cancer incidence and mortality [22, 23]. On the other hand, Nehme et al. reported that, when the activation of the system in daily practice was at the discretion of the endoscopist, it was activated in 52.1% of cases and did not improve ADR [24]. Also, Levy et al. [25] compared ADRs before and after the implementation of CADe in a high-volume center. Surprisingly, both ADR and polyp detection rate remarkably decreased with CADe. Nevertheless, AI tools are suggested as feasible performance enhancements in detecting and characterizing gastrointestinal lesions, standardization of reporting quality metrics, optimization of workflows, and aiding in predictive modeling and diagnosis [26].

The expanding implementation of CADe has led to an increasing number of young endoscopists undergoing training in an AI environment. The potential impact of training using CADe on the future performance of trainees is undefined. This study aimed to evaluate the quality indicators of endoscopists trained using AI in comparison to those trained conventionally.

## Methods and procedures

**Fig. 1:**
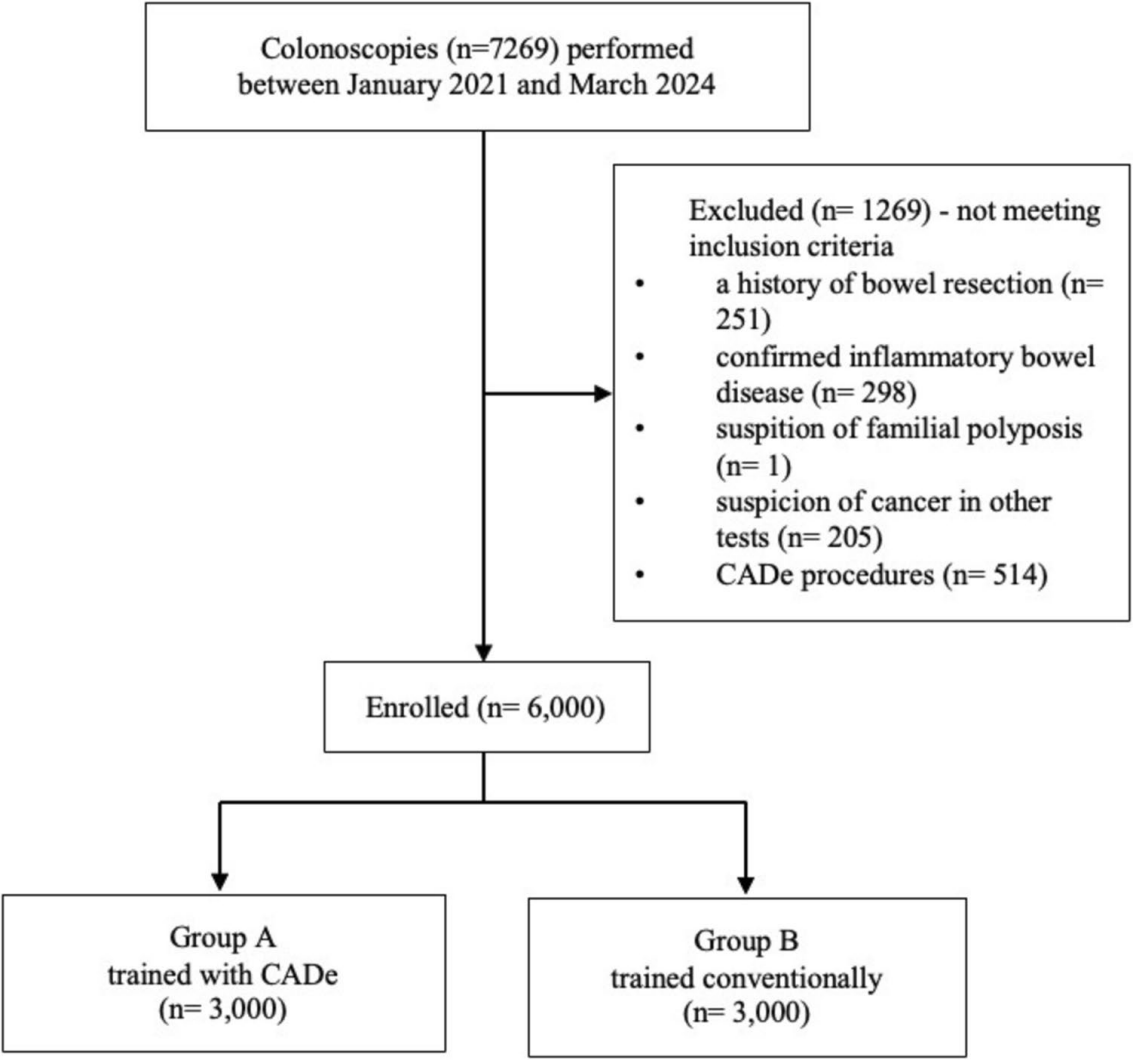
Study flow diagram. ^a^CADe: computer-aided detection

In a retrospective cohort study, we evaluated quality indicators of six endoscopists after completing training, relying entirely on endoscopists’ detection skills without AI enhancement. The endoscopy training followed the criteria established by the European Society of Gastrointestinal Endoscopy [27]. It began with a theoretical course, a one-month observership, and supplementary ex-vivo training. Subsequently, trainees were permitted to perform colonoscopies under supervision, with a minimum requirement of 500 procedures to complete the training. Three endoscopists were trained with CADe implementation, and three conventionally without additional AI assistance. After training completion, the quality indicators of young career endoscopists were evaluated in the first 1,000 examinations performed without AI enhancement to measure endoscopist’s independent performance. This resulted in a comparison between group A of 3000 patients examined by endoscopists trained in an AI environment and group B of 3000 patients examined by endoscopists trained conventionally. The study enrolled patients over 18 years old who underwent a colonoscopy for various reasons between January 2021 and March 2024 (Fig. 1). Exclusion criteria were a history of bowel resection, a confirmed inflammatory bowel disease, a suspicion of familial polyposis, a suspicion of polyps or cancer in other imaging tests, and procedures performed with CADe (after completing the training).

**Fig. 2:**
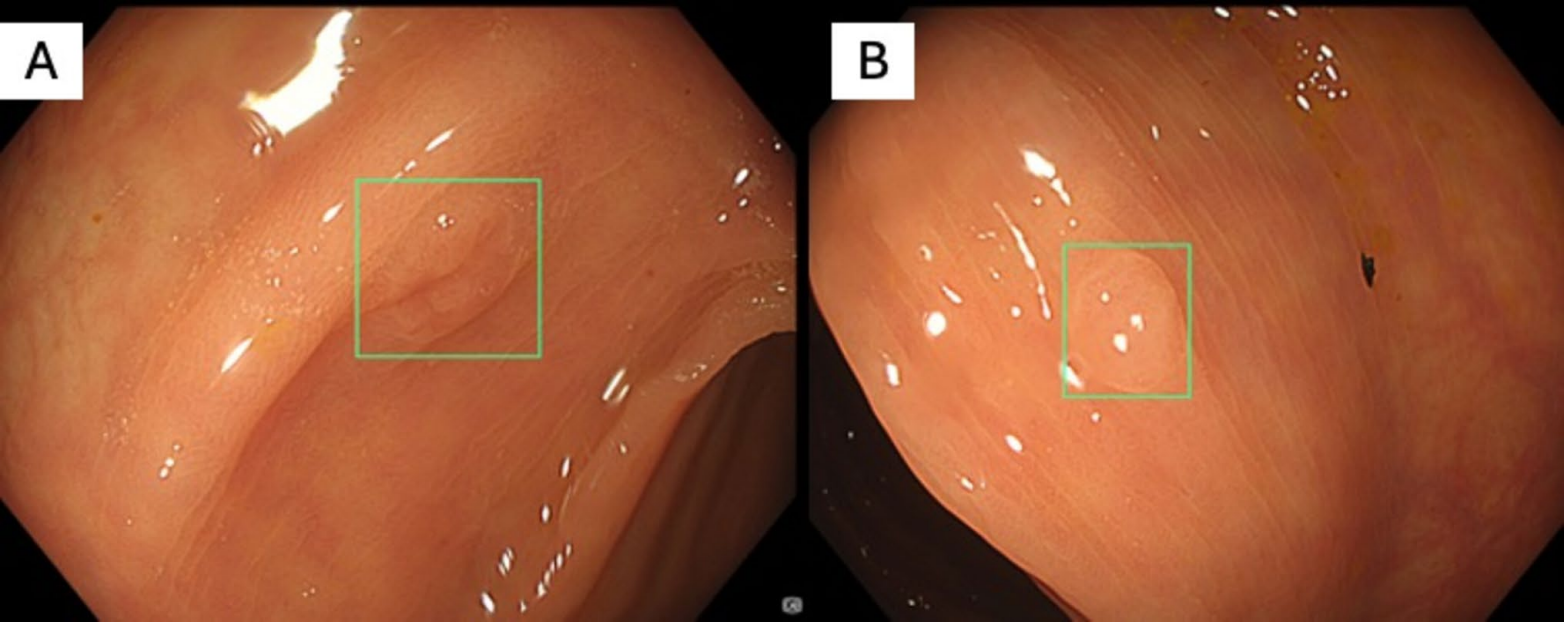
**A**, **B**: Examples of CADe implementation in the target mode. Green boxes highlight polyps in real time during colonoscopy

We used the latest Olympus X1 series endoscope set, which uses LED lighting, 4 K ultra high-resolution technology, and monitors dedicated to the system. The additional AI module that enhanced training in group A was ENDO-AID OIP-1. The system is designed to aid endoscopists in detecting polyps. The equipment is safe and presents no risks to the patient. CADe uses a complex algorithm created by machine learning, which alerts the endoscopist in real time when a suspicious lesion appears on the screen. During routine examinations, the image from the camera located in the scope is transmitted to the processor and displayed on the monitor screen. With CADe, the image from the processor is transferred to the AI-powered device. The neuronal network recognizes the shape and structure of the polyps and highlights suspicious areas on the monitor screen in real time (target mode) (Fig. 2).

Colonoscopies were conducted in an outpatient setting. Bowel preparation for colonoscopy consisted of oral administration of liquid laxatives, namely 420 g of macrogol per 4 L of water, administered in two doses (split-dose), with the last dose approximately 5 h before the exam. No additional prokinetics were used. Bowel preparation was assessed using the Boston Bowel Preparation Scale (BBPS) [28]. Adequate BBPS score was defined as at least 6 points reached. Insufflation of carbon dioxide was routinely used. Patients were initially positioned on the left side, while additional maneuvers such as patient repositioning, manual abdominal compression, instrument rotation, twisting, bracing, and extension depended on the personal preference of the endoscopist. Cecal intubation was considered complete when the ileocecal valve and the entrance to the appendix were correctly identified.

Complementary data were collected on each patient: age, gender, height, weight, and bowel preparation. Quality indicators that were assessed as primary outcomes included ADR, advanced adenoma detection rate (AADR), sessile polyp detection rate (SDR), cecal intubation rate (CIR), and withdrawal time (WT). CIR was calculated as the percentage of colonoscopies with successful cecal intubations and based on statements from colonoscopy results. WT was defined as the time from the cecal intubation to the end of the examination and based on statements from the procedural protocol, photos, or videos. The ADR, AADR, and SDR were measured as a percentage of colonoscopies when at least one adenoma, advanced adenoma, or sessile lesion were found, respectively, and based on pathology reports. The secondary outcome was the laterally spreading tumors (LST) detection rate that is the percentage of colonoscopies when the LST was found. LST was defined as a neoplasm with predominantly lateral growth of at least 10 mm in diameter, in opposition to polypoid (upward growth) or flat and depressed lesions (downward growth) [29]. The secondary assessment included the size, the Paris classification, and the Kudo classification [30, 31].

The study was conducted according to STROBE guidelines. It was based on a review of hospital records and received an institutional consent from the medical facility for data gathering without individual informed consents from participants due to the retrospective nature of the study. The study was accepted by the local ethics committee (approval number: 118.0043.1.331.2024; Ethics Committee, Jagiellonian University in Krakow) and registered on ClinicalTrials.gov (NCT06623331).

## Statistical analysis

Statistical analyses were performed using SPSS 28.0.2 software for MacOS (SPSS Inc., Chicago, IL). The missing data were reported and managed using multiple imputation method (5-times iterations, Markov Chain Monte Carlo). All continuous variables had non-normal distribution referring to the Kołmogorov–Smirnov test. Therefore, the variables were demonstrated as the median and interquartile range (IQR; 25–75%) (Table 1). The groups were compared using the Mann–Whitney test for continuous data with the abnormal distribution. Categorical variables were analyzed using the chi-square test. All statistical analyses were 2-sided, with a critical 0.05 significance level. Statistical power was calculated with post hoc analysis (CIR: 81%; WT: 100%; ADR: 98.5%; AADR: 14.5%; SDR: 100%). Logistic regression models were used to identify the effect of AI-enhanced training on high-quality performance in colonoscopy.

**Table 1:** Characteristics of the analyzed groups

	Group A (AI training) *N* = 3000	Group B (conventional training) *N* = 3000	*p* value
Participants, no	3000	3000	-
Gender, female, %	54.3	52.8	0.255
Age, yr	64 (56–70)	64 (56–70)	0.977
Height, cm	173 (166–179)	173 (168–179)	0.920
BMI^b^, kg/m2	26.7 (23.4–30.7)	27.1 (24.3–30.3)	0.140
BBPS^a^	9 (6–9)	9 (6–9)	0.467
Adequate bowel preparation, %	94.1	95.9	0.467
Indications	Primary screening: 13.2% Positive-FOBT^c^: 15.9% Follow-up: 30.6% Symptoms: 37.1% Unknown: 3.2%	Primary screening: 12.5% Positive-FOBT^c^: 17.1% Follow-up: 28.6% Symptoms: 39.0% Unknown: 2.8%	0.885

^a^*BBPS* Boston bowel preparation scale, ^b^*BMI* body mass index, ^c^*FOBT* fecal occult blood test

## Results

A total of 6000 participants were included in the overall analysis. We compared two groups of 3000 participants that were equal demographically and regarding colonoscopy indications (Table 1). The median age in both groups was 64 years. In group A, 54.3% of participants were female, compared to 52.8% in group B. Both groups had comparable height and BMI. The mean BBPS score was 9 in both groups, and adequate bowel preparation was reported in a similar percentage of cases (Table 1).

**Fig. 3:**
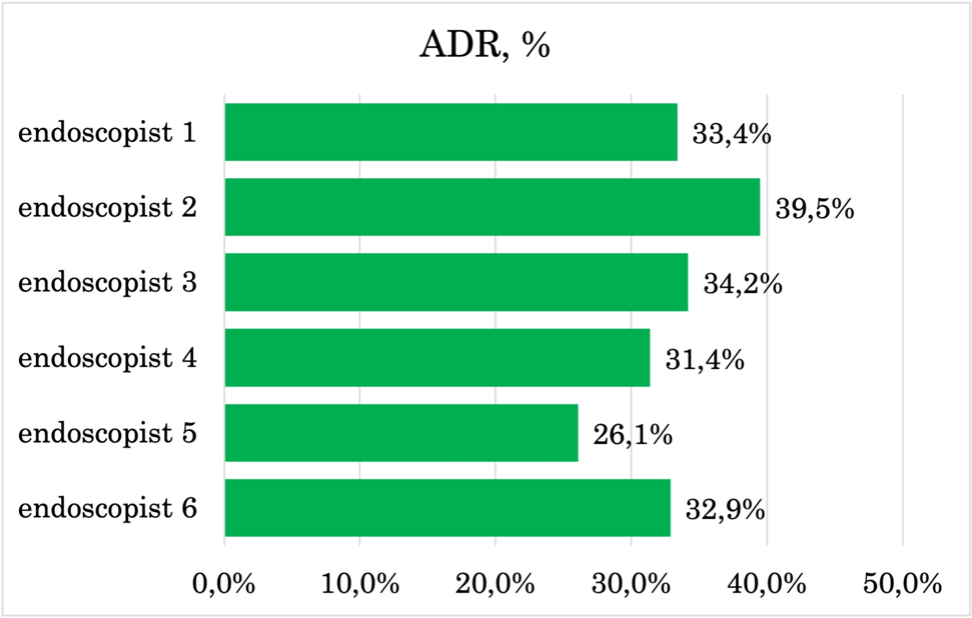
Adenoma detection rate (ADR^a^) of each endoscopist (%)

**Fig. 4:**
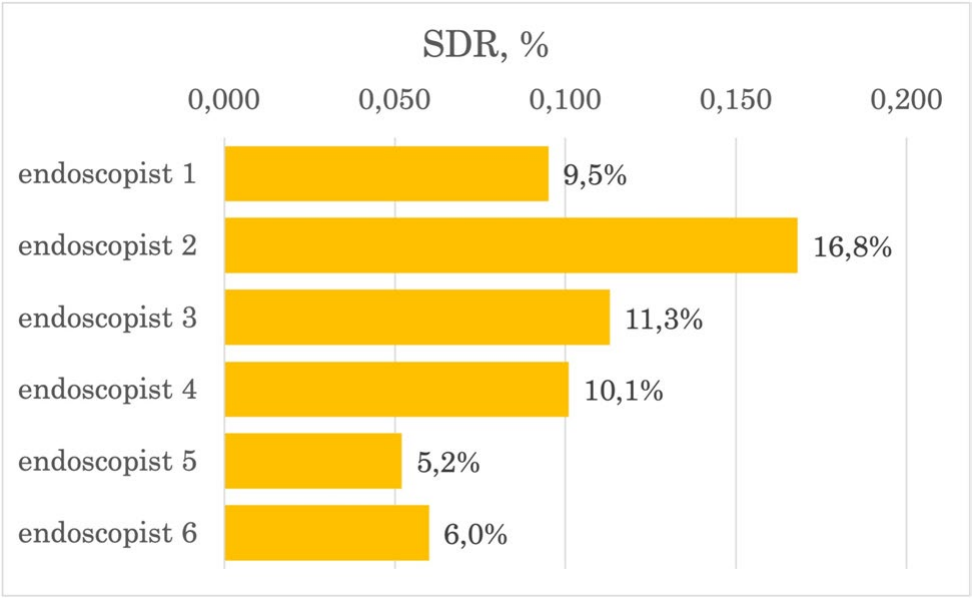
Serrated lesions detection rate (SDR^a^) of each endoscopist (%)

**Fig. 5:**
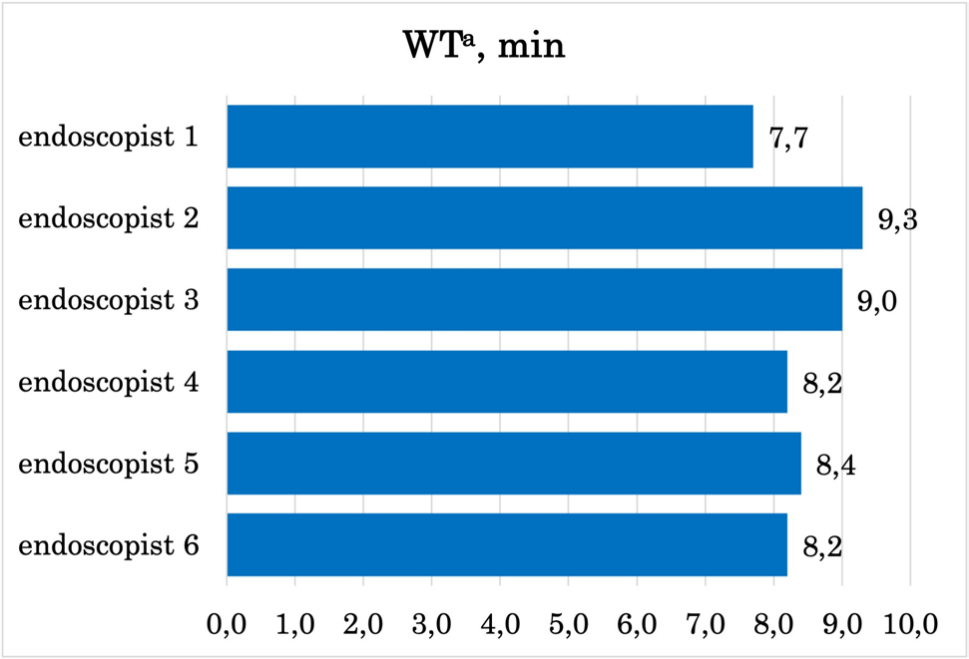
Withdrawal time (WT^a^) of each endoscopist (min)

The intraprocedural quality indicators for colonoscopy were calculated (Table 2). WT was longer in the AI-trained group (mean difference 0.8 min; 95% CI: 0.6 to 1.0 min). Groups had similar CIR (mean difference 1.5%; 95% CI: − 0.3% to 3.3%). AI-enhanced training improved ADR by 5.3% and SDR by 5.4% (95% CI: 2.9–7.6%, and 3.8–7.0%, respectively) in high-power analysis (98.5–100%). Individual WTs, ADRs, and SDRs are illustrated in Figs. 3, 4, 5. The impact on AADR was unremarkable (mean difference 0.5%; 95% CI − 0.6% to 1.6%), but the power of the analysis was low (14.5%). The missing data rates were: CIR 0%, WT 38.2% (no data on WT in the protocol, no video, or missing photos of the cecum or rectum), ADR 0.6% (polyps not retrieved), SDR 0.6% (polyps not retrieved), AADR 27% (polyps not removed, referred to another facility and lost to follow-up).

**Table 2:** Quality indicators in analyzed groups. Data quality was determined by MDR^e^ and PA^f^ (High: MDR^e^ < 5% and PA^f^ > 80%; Intermediate: MDR^e^ 5–20% or PA^f^ 40–80%; Low: MDR^e^ > 20% or PA^f^ < 40%; Very low: MDR^e^ > 5% and PA^f^ < 80%)

	Group A (AI training) N = 3000	Group B (conventional training) N = 3000	Mean difference (95% CI^c^)	Data quality
CIR^d^, %	96.3	94.8	1.5 (− 0.3 to 3.3)	High (MDR^e^: 0%, PA^f^: 81%)
WT^g^, min	9	8	0.8 (0.6–1.0)	Low (MDR^e^: 38.2%, PA^f^: 100%)
ADR^a^, %	35.5	30.3	5.3 (2.9–7.6)	High (MDR^e^: 0.6%, PA^f^: 98.5%)
AADR^b^, %	5.1	4.6	0.5 (-0.6 to 1.6)	Very Low (MDR^e^: 27%, PA^f^: 14.5%)
SDR^f^, %	12.5	7.1	5.4 (3.8–7.0)	High (MDR^e^: 0.6%, PA^f^: 100%)

^a^*ADR* adenoma detection rate, ^b^*AADR* advanced adenoma detection rate, ^c^*CI* confidence interval, ^d^*CIR* cecal intubation rate, ^e^*MDR* missing data rate, ^f^*PA* power analysis result, ^f^*SDR* sessile polyp detection rate, ^g^*WT* withdrawal time

The morphology of the polyps was assessed with a focus on LSTs detection (Table 3). No difference was reported in total rates of LSTs detection regardless of the size. Further, a separate analysis was conducted for granular (LST-G) and non-granular (LST-NG) lesions. The detection of LST-G lesions was similar in both groups regardless of the size. AI-assisted endoscopy training significantly improved the detection of LST-NG lesions smaller than 20 mm by 0.2% (CI 95%: 0.1–0.4%). No difference was noted in the detection of LSTs larger than 20 mm and overall LST-NG calculation. The missing data rate regarding LST was 0%. However, the power of the tests was low (6–41%) (Table 3).

**Table 3:** Detection of laterally spreading tumors in analyzed groups. Data quality was determined by MDR^e^ and PA^f^ (High: MDR^e^ < 5% and PA^f^ > 80%; Intermediate: MDR^e^ 5–20% or PA^f^ 40–80%; Low: MDR^e^ > 20% or PA^f^ < 40%; Very low: MDR^e^ > 5% and PA^f^ < 80%)

		Group A (AI training) *N* = 3000	Group B (conventional training) *N* = 3000	Mean difference (95% CI^a^)	Data quality
LST-G^c^	10-20 mm	9 (0.3%)	6 (0.2%)	0.1% (-0.2% to 0.4%)	Low MDR^e^: 0%, PA^f^: 12%
	> 20 mm	12 (0.4%)	12 (0.4%)	0.0% (-0.3% to 0.3%)	Undetermined MDR^e^: 0%, PA^f^: -
	overall	21 (0.7%)	18 (0.6%)	0.1% (-0.3% to 0.5%)	Low MDR^e^: 0%, PA^f^: 7%
LST-NG^d^	10-20 mm	10 (0.3%)	3 (0.1%)	0.2% (0.1% to 0.4%)	Intermediate MDR^e^: 0%, PA^f^: 41%
	> 20 mm	12 (0.4%)	14 (0.5%)	0.1% (-0.3% to 0.4%)	Low MDR^e^: 0%, PA^f^: 8%
	overall	22 (0.7%)	17 (0.6%)	0.2% (-0.2% to 0.6%)	Low MDR^e^: 0%, PA^f^: 7%
Total LSTs^b^	10-20 mm	19 (0.6%)	9 (0.3%)	0.3% (-0.1% to 0.7%)	Intermediate MDR^e^: 0%, PA^f^: 41%
	> 20 mm	24 (0.8%)	26 (0.9%)	0.1% (-0.3% to 0.5%)	Low MDR^e^: 0%, PA^f^: 6%
	overall	43 (1.4%)	35 (1.2%)	0.3% (-0.3% to 0.8%)	Low MDR^e^: 0%, PA^f^: 10%

^a^*CI* confidence interval, ^b^*LSTs* laterally spreading tumors, ^c^*LST-G* laterally spreading tumor – granular type, ^d^*LST-NG* laterally spreading tumor – non-granular type, ^e^*MDR* missing data rate, ^f^*PA* power analysis result

Logistic regression confirmed that AI-trained endoscopists were more likely to meet high-quality standards in terms of ADR, SDR, and WT (OR 1.27, 95% CI: 1.14–1.42, *p* < 0.05; OR 1.93, 95% CI: 1.10–3.37, *p* < 0.05; OR 1.88, 95% CI: 1.69–2.09, *p* < 0.05, respectively), with no impact on CIR (Table 4).

**Table 4:** The effect of AI-enhanced training on high-quality performance in colonoscopy

	OR^e^	95% CI^b^	*p* value	Data quality
CIR^c^	1.20	0.95–1.52	0.132	High (MDR^d^: 0%)
ADR^a^	1.27	1.14–1.42	< 0.05	High (MDR^d^: 0.6%)
SDR^f^	1.93	1.10–3.37	< 0.05	High (MDR^d^: 0.6%)
WT^g^	1.88	1.69–2.09	< 0.05	Low (MDR^d^: 38.2%)

Data quality was determined by MDR^e^ (High: < 5%; Low: > 5%)

^a^*ADR* adenoma detection rate, ^b^*CI* confidence interval, ^c^*CIR* cecal intubation rate, ^d^*MDR* missing data rate, ^e^*OR* odds ratio, ^f^*SDR* sessile polyp detection rate, ^g^*WT* withdrawal time

## Discussion

Colonoscopy is a widely recognized screening tool for detecting colorectal cancer, with its efficacy evaluated by quality indicators such as CIR, ADR, SDR, and WT [3, 4]. However, a major limitation of this procedure is the insufficient detection of suspicious lesions [5]. Cognitive errors, where the lesions are observed but not recognized as polyps by the endoscopist, are the most common cause of visual recognition errors [32]. To enhance ADR and other quality metrics, technological advancements are increasingly necessary. The development of next-generation endoscopes with 4 K resolution and CADe systems powered by AI represents a promising advancement in colonoscopy [17, 33, 34]. These technologies aim to enhance detection rates and support precise optical diagnoses. For instance, Tiankanon et al. [21] noted that CADe positively impacted ADR, even among experienced endoscopists with already high detection rates. On the other hand, Patel et al. [35] found no significant difference in ADR between CADe-assisted and standard colonoscopy in the meta-analysis of nonrandomized real-world studies. Despite these mixed findings, AI tools are widely regarded as transformative, with 95.5% of endoscopists believing they will positively impact their practice [36]. As AI becomes a standard feature in endoscopic equipment, an increasing number of young endoscopists are being trained in AI-enhanced environments. This study evaluated AI’s role in endoscopy training and its influence on trainees’ performance in future procedures. We analyzed 1,000 colonoscopies performed by early-career endoscopists allowing us to assess their performance at the same professional stage.

The endoscopy training requires extensive practice and the access to high-quality facilities. Notably, ADR was found to range from 14 to 36% among gastroenterology trainees. According to van Doorn et al., this variance persists, and ADR does not improve after training [37]. Biscaglia et al. [38] conducted a tandem study revealing that CADe effectively closed the detection gap between trainees and experts, with ADRs of 38% and 40%, respectively (*p* > 0.05). Thus, CADe demonstrates its ability to standardize detection quality and support optical diagnoses across a diverse range of practitioners. A meta-analysis by Aziz et al. [39] highlighted the benefits of dual observer strategies, where additional observers such as nurses, trainees, or technicians helped achieve significantly higher ADRs compared to just one endoscopist. The CADe system acts as a “virtual second observer” during endoscopy training, although the impact of such training on future quality metrics was unclear [40].

International guidelines establish quality standards for colonoscopy procedures [3, 4]. For instance, a minimum ADR of 25% is recommended [3, 4], and recent data suggest that achieving 35% or higher significantly reduces interval colorectal cancer risks [7, 41]. In our study, AI-enhanced training increased ADR by 5.3%. Both groups reached the minimum ADR, but only the AI-trained group exceeded 35% (Table 2) [3]. AI-trained endoscopists also surpassed aspirational SDR threshold of 10%, achieving an optimal level of 12.5%, compared to 7.1% in conventionally trained practitioners. Additionally, WT minimum is 6 min, with aspirational targets of 9 min (American Gastroenterological Association) or 10 min (European Society of Gastrointestinal Endoscopy) [3, 4]. Huang et al. reported higher ADR and polyp detection rate when trainees’ WTs exceeded 8 min [42]. In our study, conventionally trained endoscopists averaged 8 min WT, meeting good-quality standards, while the AI-trained group averaged 9 min. This improvement likely results from AI-driven habits of detailed colon evaluation, reinforced by the frequent alerts provided during training. However, the WT data quality was deemed low due to high missing data rate, and conclusions should be drawn with caution.

Furthermore, we recognized AI-enhanced training as a factor of high-quality performance in terms of ADR and SDR (Table 4). Thus, AI-trained endoscopists were almost twofold more likely to achieve high SDR. These results underscore the superior performance of AI-enhanced training, with group A exceeding aspirational targets, while group B met only minimum quality standards. The most noticeable differences in individual performance were observed in ADR and SDR, where the gaps between the highest and lowest performers were the most pronounced (Fig. 3, 4). These disparities highlight the enhancement of cognitive skills through AI training.

Certain colorectal lesions, such as those with depressed components, LST-NGs, and mixed-type LSTs, pose a higher malignancy risk [43, 44]. LSTs, defined as superficial neoplasms ≥ 10 mm [45], grow laterally rather than vertically, and their morphology often makes detection challenging. Wang et al. noted that small, isochromatic, flat lesions with unclear boundaries are frequently missed during single colonoscopies [46]. Non-granular serrated lesions are particularly difficult to detect due to their similarity to normal mucosa. Our findings indicate that AI-trained endoscopists had higher detection rates for LST-NG smaller than 20 mm, while detection rates for larger lesions or granular LSTs showed no significant differences. However, power of the analysis was low. Much larger sample is required to establish differences in LST detection.

Several studies have addressed quality indicators in endoscopy training and highlighted factors that influence trainee performance. Schult et al. [47] reported that trainees achieved high-performance indicators, including ADR and CIR, and, with time, withdrawal times were reduced without compromising ADR. On the other hand, Eckard et al. [48] assessed detection rates of supervised trainees and experts and reported that trainee ADR was reduced by 4.4% (19.3% vs. 14.9%) and AADR by 3.7% (8.6% vs. 4.9%). However, a follow-up study by the same author found no significant difference in ADR during screening exams (19.5% vs. 21.4%; *p* = 0.19) [49]. Further, Say et al. [50] observed that trainee involvement reduced SDR but had minimal impact on other metrics. These findings underscore the importance of structured training and monitoring quality indicators to ensure early-career endoscopists achieve high performance. Structured training programs and evaluation systems have proven effective in mitigating variability in outcomes, often attributed to behavioral habits [51, 52].

Mentorship plays a critical role in enhancing trainee performance, as crucial skills are passed on to trainees from their mentors [53]. Mahadev et al. [54] demonstrated that trainees achieved higher ADR when supervised by high-performing mentors. Given that ADR performance rarely improves after training, early intervention is essential [37]. Fellows participating in structured mentorship programs consistently outperformed other peers during conventional colonoscopy training [55]. In our study, CADe systems appeared to replicate the benefits of mentorship, improving trainee performance during training and achieving exceptional outcomes.

Feedback has also been identified as a key factor in improving outcomes. Constructive and timely feedback has been shown to accelerate skill acquisition. Trainees who received feedback achieved faster cecum intubation and improved mucosal visualization compared with those who did not [56]. Kaltenbach et al. [57] compared video-based feedback with conventional feedback in cold snare polypectomy training and found that video-based feedback significantly enhanced trainee competence, despite a long learning curve. Van Doorn et al. [37] emphasized the importance of early benchmarking and feedback to improve ADR in future practice. Their research demonstrated that low-performing trainees rarely improved ADR after training, with ADR among gastroenterology trainees ranging from 14 to 36%. Klare et al. [58] also highlighted performance variability, with trainee ADRs ranging from 17 to 31%. These findings underline the importance of implementing targeted feedback and training interventions early in a trainee’s development to address gaps in performance. Similarly, CADe systems, by providing real-time feedback during procedures, may instill habits of thorough colon evaluation, positively impacting performance even when AI tools are not utilized. In fact, the high number of false-positive results in real-time on-screen reports, often cited as a limitation of CADe, may actually serve as a trigger for increased awareness and have a beneficial influence on the performance of early-career endoscopists, as observed in our study. Trainees who are required to verify the alerts may develop a more precise technique, leading to higher-quality performance. However, an improved performance following the introduction of AI may not apply to experts, as suggested by previous reports [24, 25]. This highlights the differing cognitive impacts of external support from technological advancements on early-career and expert endoscopists.

AI-powered systems are increasingly recognized as valuable tools in endoscopy training. One promising approach involves AI-powered feedback during simulated colonoscopy, which has been shown to enhance trainee performance and reduce patient-related risks [59, 60]. Simulator-based training effectively develops essential skills and facilitates their application in clinical practice [61]. Skills acquired through proficiency-based virtual reality simulator training can be retained for several months [62]. Koch et al. demonstrated that intensive simulator training early in the learning curve significantly improved performance in patient-based colonoscopies [63]. However, while virtual-based training improves early skill acquisition, it is not a replacement for conventional training methods. Instead, it should be used as a supplement [64]. Feedback remains an integral part of simulator-based learning, ensuring trainees achieve proficiency [65, 66]. However, evidence on the influence of CADe on training outcomes is limited. Although Yamaguchi et al. reported that while CADe systems did not directly improve ADR, they significantly reduced adenoma miss rate and enhanced trainees’ ability to accurately locate colorectal adenomas [67]. In our study, CADe appeared to enhance performance, developing habits of detailed colon evaluation that positively influenced future procedures.

The initial training phase is a crucial period in an endoscopist’s career, as its quality directly impacts future performance and equips practitioners with essential skills for effective and proficient practice. The new generation of endoscopy practitioners can benefit from the latest technological advancements. Although current evidence on the impact of CADe and simulator-based training is still evolving, technological advancements are transforming endoscopy training. This study underscores the potential of these tools to enhance training outcomes and highlights the need for further research into their long-term impact on trainee performance. However, the full potential of AI in endoscopy training requires further investigation through prospective randomized controlled trials.

## Strengths and limitations

The presented findings confirm the positive impact of AI-enhanced training on trainees’ future quality measures, as demonstrated by a large sample analysis. The study design ensured a comparison of the performance at the same point of the career. All endoscopists achieved at least the minimum required quality performance levels. Analyses of major quality indicators (ADR, SDR) had high power and low missing rates.

However, it is imperative to acknowledge certain limitations inherent in the study. First, the evaluation was retrospective and based solely on single-center clinical data. The data on WT and AADR were of low quality due to high missing rates and required the application of a multiple imputation model. The AADR and LSTs analyses had low power. Additionally, only six early-career endoscopists were included in the analysis, and their training methods may have varied due to the involvement of multiple mentors. Furthermore, no additional evaluation of endoscopists’ performance was conducted and there were no data on their ADR during training. Some indications for colonoscopy were unclear among participants. Lastly, there was no structured evaluation of colonoscopy-related complications and long-term outcomes, including PC-CRC and interval cancer.

## Conclusions

Implementing CADe during endoscopy training may positively impact quality indicators in the first 1000 cases. Endoscopists trained in an AI-enhanced environment exceeded the aspirational targets of the quality guidelines, whereas those trained conventionally achieved only the minimum required quality measures. AI-enhanced training was identified as a factor of high-quality performance in terms of ADR, SDR, and WT. Novel technologies and innovations may improve early-career endoscopists’ performance. However, the full potential of AI in endoscopy training requires further investigation through prospective randomized controlled trials.
